# Advanced molecular poliovirus diagnostics across multiple real-time PCR platforms with locked nucleic acids and primer overhangs

**DOI:** 10.1128/jcm.00658-26

**Published:** 2026-09-16

**Authors:** Nancy Gerloff, Hong Sun, Brandon Short, Eric Harvey, Stacey Jeffries-Miles, Paula L. Marcet, Katherine D. Poston, Christina J. Castro, William A. Nix, Muhammad Masroor Alam, Shelina Moonsamy, Maël Bessaud

**Affiliations:** 1 Division of Viral Diseases, U.S. Centers for Disease Control and Prevention 1242, Atlanta, Georgia, USA; 2 Contracting Agency to the Division of Viral Diseases, Tanaq, Atlanta, Georgia, USA; 3 National Polio Laboratory, National Institute of Health, Islamabad, Pakistan; 4 A Division of the National Health Laboratory Service, National Institute for Communicable Diseases 70687, Sandringham, South Africa; 5 Institute Pasteur 27058, Paris, France; St Jude Children's Research Hospital, Memphis, Tennessee, USA

**Keywords:** real-time RT-PCR assay, locked nucleic acid, assay development, primer overhangs, wild-type 1 poliovirus, poliovirus eradication, vaccine-derived polioviruses, real-time PCR probe design

## Abstract

This study details the development and validation of five real-time RT-PCR assays for the intratypic differentiation (ITD) of polioviruses and the discrimination of vaccine-derived polioviruses (VDPVs) utilizing locked nucleic acid (LNA) technology and primer overhangs. Updated assays, including wild-type 1/Qβ duplex (WPV1), VDPVs 1 and 3, wild-type 3, and novel oral poliovirus type 2 (nOPV2), were designed in response to challenges posed by genetic diversity and detection gaps in existing protocols following outbreaks, and the introduction of a new vaccine replacing the Sabin 2 oral poliovirus vaccine (OPV). The CDC’s Polio and Picornavirus Branch led assay development, supporting global surveillance efforts with kits tailored for use across multiple real-time PCR platforms. Comprehensive virus collections representing all three poliovirus serotypes, nonpolio enteroviruses, and vaccine seed strains were used in the validation. Assay performance was assessed through piloting in poliovirus reference laboratories across outbreak regions, including Central Asia and Africa. Combining primers with AT-rich overhangs enhanced fluorescence, improving interpretation of weak signals for VDPV1 and WPV1 assays, and boosting analytical sensitivity for the VDPV3 assay by 10-fold to 100-fold (100%). The new assays enabled reliable detection of extinct WPV3 (100% sensitivity and 96.97% specificity), the circulating genotype WPV1 (100% sensitivity and 98.72% specificity), and discrimination of Sabin 1 and 3 from vaccine-derived strains, and the discrimination of nOPV2 from Sabin 2 (100% specificity) from surveillance samples. These advancements mark a significant milestone in molecular diagnostics for poliovirus eradication, supporting surveillance and response efforts worldwide, with remaining pockets of circulating VDPVs and wild-type 1 viruses.

**IMPORTANCE:**

Recent poliovirus detections from wastewater in non-endemic regions sparked renewed interest in the agent. Limited access to extinct polioviruses for validation studies hinders diagnostic laboratories from designing and validating real-time RT-PCR assays for specific and sensitive detection in wastewater or stools. There are currently no commercial tests that can detect and type all poliovirus serotypes that have been assessed with recent endemic or seed viruses used by vaccine manufacturers, making the intratypic differentiation unique. This work describes the redesign and validation of the five RT-PCR assays with various poliovirus strains such as vaccine seed strains, outbreak strains, and eradicated viruses. Locked nucleic acids and primer overhangs were applied to the primer and probe design, resulting in advanced laboratory capability to detect contemporary poliovirus strains, strengthening surveillance in support of the global poliovirus eradication activities.

## INTRODUCTION

Wild poliovirus (WPV) has been declared eradicated in all countries except for type 1 in Afghanistan and Pakistan, where it is still endemic (1). The Global Polio Eradication Initiative (GPEI) has been tasked with the surveillance of acute flaccid paralysis (AFP, roughly 220,000 specimens per year) and environmental surveillance (about 15,000–20,000 wastewater specimens per year) (2). The molecular assays for detection and screening of cell culture isolates are a suite of assays called intratypic differentiation (ITD) of polioviruses; a kit, based on real-time reverse transcription PCR (rRT-PCR) in a one-step format. The ITD suite is a screening PCR test detecting enterovirus (PanEV), Sabin 1, 2, and 3 (Quadruplex EV/Sabin assay), any poliovirus (PanPV), wild-type 1 (WPV1), poliovirus type 2 (PV2), novel oral poliovirus vaccine type 2 (nOVP2), and wild-type 3 (WPV3) assays.

The vaccine-derived poliovirus (VDPV) assays, which form part of a reflex testing algorithm, discriminate oral poliovirus vaccine strains (OPV, Sabin 1 and Sabin 3) from genetically drifted VDPVs, which have the potential to cause an increase in AFP cases. The two VDPV real-time assays are inverse detection tests with the absence of a signal (no CT or negative) indicating mutation(s) in the antigenic site of Sabin 1 or Sabin 3 interrupting probe binding in the assay(s) (3). It is intended to identify any poliovirus isolate of programmatic importance, which is then forwarded to confirmatory sequencing. The suite of assays protects against pipetting errors or viral genetic drift by redundant assay targets, including any poliovirus genotype. The Polio and Picornavirus Branch at the CDC provides real-time testing kits for about 134 of the Global Polio Lab Network (GPLN) laboratories and handles large-scale kit production with ATCC, a contracting agency to the CDC (4–6). In the past years, most outbreaks have been attributed to Type 2 VDPV2, but several VDPV1 and a few VDPV3 outbreaks were reported from regions where OPV is used for immunization (7).

The design goals were to reduce the number of assays (WPV3), increase specificity for outdated assays (VDPV1 and WPV1), increase sensitivity for VDPV3, and be able to detect and discriminate the vaccine strain (nOPV2) from Sabin 2. The last updates to the ITD assays were reported by Sun et al. in 2021, describing the improved probe in the PanPV assay, followed by Miles et al., who reported on the WPV1 assay duplexed with Qβ bacteriophage to include nucleic acid extraction controls in the testing algorithm (5, 6). The ITD version 5.0 (issued in 2018) contained two separate WPV type 3 assays (WPV3-I and WPV3-II) targeting two genotypes that had been declared eradicated in 2019 (WPV3 genotypes were based on their last occurrence in the African continent and in Southeast Asia) (8). The new WPV3 assay needed to detect both genotypes with very large sequence diversity while maintaining its sensitivity and high specificity. The duplex WPV1/Qβ assay needed to be updated after a genetically diverse genotype of wild-type 1 PV started to circulate in Afghanistan and Pakistan in 2020 that was sometimes missed by the WPV1 assay and showed significant cross-reactivity with an assay that detected any poliovirus type 2 (PV2 assay) (9).

VDPVs type 1 and type 3 are defined as having >1% nucleotide (nt) sequence divergence (i.e., ≥10 nt substitutions) from Sabin strains in the ~900 nt capsid protein region VP1 (10). The VDPV reflex assays distinguish Sabin-like vaccine viruses from VDPVs for types 1 and 3 (3). In 2018, Papua New Guinea observed an outbreak of VDPV, and several isolates were not identified as VDPV’s by version 4.0 of the VDPV1 assay (used between 2014 and 2018) (11). Sequencing of the VP1 region confirmed that the vaccine virus had drifted to a virulent phenotype by a few nucleotides, thereby reducing the detection sensitivity of the VDPV1 assay.

Novel OPV (nOPVs) developed for emergency use listing (EUL) necessitated the development of an assay to distinguish OPV2 (Sabin 2) from nOPV2 vaccines. Nearly 1 billion doses of nOPV2, a comparably safe, effective, but more genetically stable version of the existing OPV2, had been administered in 35 countries by December 2023, only 2 years after its release. At that time, the vaccine was granted WHO prequalification, marking the end of its rollout under the WHO EUL procedure, becoming the first-ever vaccine to graduate from EUL to prequalification. The nOPV2 assay was designed to be run in parallel with the ITD suite and included in the ITD version 6.0 poliovirus diagnostic algorithm (12, 13).

This study describes the design and performance of five real-time RT-PCR assays in the ITD of polioviruses and reflex assays for the discrimination of vaccine viruses (Sabin 1 and 3) from VDPV using locked nucleic acid (LNA) technology and primer overhangs (AT-rich 12-mer) (14–16).

The updated assays (WPV1/Qβ duplex, VDPV1 and 3, WPV3, nOPV2) were validated in-house and piloted across multiple GPLN laboratories. There were various considerations for the design of the ITD or VDPV assays that had to be met: (i) the new assays’ ability to detect with specificity and high sensitivity and not be inferior to the existing assays, (ii) the ability to keep the same run method for all assays, and (iii) perform the PCR in any of the real-time PCR cyclers the network uses (i.e., Thermo Fisher ABI7500, Quant Studio 5, Bio-Rad CFX 96 or CFX Opus, etc.) (17). The new sets of primers and probes contained in ITD version 6.1 and VDPV version 6.0 assay kits provide higher sensitivity and work well with extracted RNA as well as isolated viruses in different PCR platforms used in the GPLN laboratories. Our new assay suite marks another milestone for the diagnostic assay development CDC has undertaken over the past 5 years.

## MATERIALS AND METHODS

### Reference virus collection

Real-time RT-PCR assays were evaluated against viruses from the unique CDC collection representing the three PV serotypes, nonpolio enteroviruses (NPEV, non-typed), CPE-positive, and CPE-negative culture supernatants. Viruses were isolated in L20B cells (recombinant murine cells that express human poliovirus receptor [PVR]) and human rhabdomyosarcoma cells (RD; ATCC cat. no. CCL-136) after inoculation of stools from AFP or contact cases that were collected from 1999 through 2025 by laboratories in the GPLN globally (Table S1) (18–21). The composition of the virus testing panel was (*n* = 184): Sabin 1 (*n* = 10), VDPV1 (*n* = 11), Sabin 3 (*n* = 12), VDPV3 (*n* = 14), Type 2 (WPV2, VDPV2, and Sabin 2, *n* = 41), WPV1 (*n* = 37), WPV3 (*n* = 33); NPEV (*n* = 15) (coxsackievirus A4 [CV-A4], CV-A9, CV-A21, CV-B4, and CV-B5, echovirus 6 [E-6], E-9, E-11 [*n* = 2], E-12, enterovirus A71 [EV-A71], EV-D68, EV/rhinovirus, rhinovirus A47, and unknown NPEV); and negative for enteroviruses (*n* = 11). Type 3 viruses belonged to WPV3 genotype WEAF-B3 (West Africa B3, *n* = 13), SOAS (South Asia, *n* = 20); and non-target-specific Sabin 3-like isolates (*n* = 12); VDPV3 isolates (*n* = 14) and mixtures of Sabin 3/PV2 and WPV3/PV2 (PV type 3 mixtures). In the nOPV2 assay development, nOPV2 (GenBank acc. no. MZ245455, Table 1), MEF-1 (PV type 2 IPV production; acc. no. AY238473.1), and Sabin 2 reference strain (OPV2, acc. no. AY184220) were used in addition to the nOPV2 (*n* = 21) and Type 2 viruses mentioned above (*n* = 41) (12). Non-containable, attenuated virus strains, S19-Sabin 1, 2, and 3, S19-Mahoney (type 1), S19-MEF-1 (type 2), S19-Saukett (type 3) (GenBank acc. no. PP068131–PP068136), nOPV1, and nOPV3 were tested to establish the assay performance (22, 23). All poliovirus assays are performed with 1 µL of clarified virus supernatant as the template in the PCR mix.

**TABLE 1:** Limit of detection for poliovirus real-time RT-PCR ITD 6.1 assays (new assays only) and both VDPV 6.0 assays established with standardized virus isolates from vaccine strains and plaque-purified viruses

Assay name	Gene target	Polio reference virus strain	Accession number	LOD 95% (CCID_50_/µL^*a*^)	Average Ct -lowest dilution of 19/20 replicates detected (Ct range)
WPV1/Qβ duplex	WPV1	SOAS WPV1^*b*^	PX448531 ^*b*^	10	32.5 (31.9–33.1)
WPV3	WPV3	AFR-WEAF-B3	KY941933	10	32.3 (30.8–33.6)
		SOAS WPV3	KY941935	10	32.9 (30.8–34.8)
nOPV2	nOPV2-1b	nOPV2-1b	MZ245455	0.1	33.8 (32.7–36.2)
VDPV1	Sabin 1	Sabin 1	AY184219	10	33.7 (31.9–36.1)
VDPV3	Sabin 3	Sabin 3	AY184221	1	31.3 (30.6–32.7)

^*a*^ Pfu/µL: plaque-forming unit per microliter or CCID_50_/µL.

^*b*^ The isolate that was sequenced in this study.

### Plaque purification and quantification standards

The emergent WPV1 from the endemic region (WPV1-2024) was plaque purified using an adaptation of previously published methods (4). Briefly, HeLa cell monolayers (ATCC cat. no. CCL-2) were seeded into separate 10 cm culture dishes and inoculated with virus dilutions from 10^−2^ to 10^−8^. The infected cell monolayers were overlaid with 0.9% agarose in 2% complete minimal essential medium (MEM; Thermo Fisher Scientific, Waltham, MA). The solidified agarose-cell monolayers were incubated at 37°C for 48 h–72 h. Between 10 and 20 plaques were harvested into 0.5 mL MEM and subjected to three freeze-thaw cycles. Virus clones were propagated in RD cells at 37°C for 24 h, until 100% CPE was observed. Supernatants were harvested and stored at −80°C until use. To verify the identity of the plaque-purified viruses, whole-genome sequencing was performed as adapted from previously described methods (24, 25). Briefly, viral RNA was extracted with the QIAamp viral RNA minikit following the manufacturer’s instructions (Qiagen, Germantown, MD). The amplification method used here was SuperScript Shotgun; the next-generation sequencing kit was Illumina XT, with IDT indexing (129-224), standard flow cell, and 500 v2 cycles on the Illumina MiSeq instrument. We determined the lower limit of detection (LLOD) of ITD 6.1 and VDPV 6.0 assays using quantified plaque-purified poliovirus representing three serotypes, including two WPV3 genotypes (West Africa, South Asia). Viral isolates were standardized from stocks with starting titers of 10^8^ CCID_50_/mL (nOPV2), WPV1-SOAS-2024 (concentrations between 5 × 10^9^ CCID_50_/mL and detection limits) and WPV3 (10^8^ CCID_50_/mL to detection limits) (Table 1).

### Standardization of RNA transcripts

Synthetic RNA transcripts were created from published sequences (Table 1). The tandem RNA transcript was constructed with the new WPV1 genotype viral protein 1 (VP1) sequence added to the Qβ bacteriophage sequence with methods described before (4, 6). Briefly, plasmids were purchased from GenScript (Piscataway, NJ) and used to generate RNA transcripts, which were quantified in the Qubit (Thermo Fisher). After *Mlu*I enzymatic linearization of the plasmids, RNA was synthesized with HiScribe T7 High Yield RNA Synthesis Kit (New England Biolabs, Ipswich, MA). Transcripts were treated with DNase to remove excessive DNA contamination, and the quality of the transcripts was assessed with the Perfecta PCR enzyme kit (Quanta Biosciences, Beverly, MA). A specific real-time PCR for the target was run to confirm the removal of DNA to concentrations of 1 ng per reaction (10-fold dilutions starting with 1 ng to 0.01 pg, i.e., 1 µL template input in each PCR) with concentrations determined spectrophotometrically by NanoDrop (Thermo Fisher). RNA transcripts of MZ245455 were generated using the same principle as for the WPV1 VP1 construct (12, 26). Transcripts included in the real-time kits as positive controls (Sabin/EV, WPV1, WPV3, nOPV2, Sabin 1/3) are available upon request for research use from CDC.

### Real-time RT-PCR assay design

For all assays, the PCR reagent mix contains 10 µL of XLT Tough Mix PCR enzyme mix (Quanta Biosciences), 1 µL of the assay primer and probe mix (provided by ATCC, Manassas, USA, through the IRR contract with CDC), and RNAse-free water for a final reaction volume of 20 µL per reaction. One microliter of template, either clarified virus isolate or RNA-positive template control (RNA transcript), is added. The primers and probe mixes in the ITD 6.1 kit were Quadruplex Sabin/EV (targets EV, Sabin 1, 2, and 3), PanPV, WPV1/Qβ duplex, PV2, WPV3, and nOPV2 assay. The two reflex assays in the VDPV 6.0 kit are the VDPV1 (targets Sabin 1) and VDPV3 (targets Sabin 3) assays. All oligonucleotides were synthesized by Qiagen and purified by HPLC (probes > 98% purity) and standard desalting (primers > 95% purity), applying company GMP/IVD standards (Qiagen, MA). To validate the new methods, all assays were run on the compatible real-time PCR cyclers for ITD and VDPV, the CFX96 (Bio-Rad) and Applied Biosystems 7500. After full validation, additional instruments were also tested, including Rotor Gene Q, Quant Studio 5 (QS5), QS 6 flex, and Bio-Rad Opus 96. The run methods are published before; briefly, the reverse transcription step was 30 minutes at 50°C followed by 1 minute at 95°C, the PCR was 40 cycles of 95°C for 15 seconds, 50°C for 45 seconds, 72°C for 5 seconds with reduced ramp rate to 25% at the 50°C step in the 7500, and 0.4°C on QS5 and 6 (4). All experiments involving non-infectious RNA and non-containable isolates (Sabin 1, 3 strains, S19) were performed at biosafety level 2 (BSL-2), and any containable and infectious strains were tested exclusively in BSL-3. The efficiency of each assay was estimated using replicates of 10-fold dilutions of control RNA transcripts (starting with concentrations of 0.1–1 pg, down to the limit of detection) and quantified virus isolates.

### Sequencing and data availability

VP1 sequences from poliovirus isolates and RNAs collected and previously tested at CDC or from VP1 sequences provided through collaborators were used in the design of the assays (i.e., 2018–2024 WPV1-SOAS sequences, VDPV1, and historic 2009–2012 WPV3). Reference-based genome assemblies were performed in Geneious version 11 (https://www.geneious.com/), and the plug-in tool MAFFT align (MAFFT v7.490) was used to perform multiple sequence alignments of consensus genomes obtained (27). The full-length genome for the WPV1 (length 7,442 nucleotides), derived from a plaque-purified clone isolated in cell culture, has been submitted to NCBI’s GenBank database under accession number PX448531, with the post-processed trimmed sequencing reads deposited in the NCBI’s Sequence Read Archive (SRA) under BioProject PRJNA1510675 and Run Accession SRR SRR40101245 (28).

### Statistical analysis

Cycle threshold (CT) values were recorded for all individual replicates for each target assay to determine sensitivity and specificity. The no-template controls (NTC) were used to manually set the zero baseline fluorescence emissions. Cycle thresholds were used only for qualitative determination: positive (CT between 1 and 40) or negative (no CT). The lower limit of detection was calculated from the number of positive samples out of 20 replicates for all templates used in the assays. The assay CT values were averaged, and the standard deviation was used to calculate statistical significance in a two-tailed *t*-test. The CCID_50_ titer for standardized viruses was determined using the Spearman-Karber formula (29, 30).

## RESULTS

### Development of a duplex WPV3 assay with locked nucleic acids

The two WPV3 assays (genotypes WPV3-AFRO and WPV3-SOAS) underwent a name change to WPV3-I and WPV3-II, but primer and probe sequences remained unchanged, and there was a need to reduce the overall number of assays run in parallel (six assays in the ITD version 5.0) (4). The WPV3-II assay had undesired cross-reactivity with Sabin 3 viruses and background fluorescence that was hard to interpret. The challenge in designing the assay was overcoming a high sequence diversity within clades and between the two genotypes (sequence identity as low as 83% in one genotype and higher between genotypes). To derive primers and probes, a consensus sequence was created in Geneious from a large collection of genetically diverse WPV3 VP1 sequences (*n* = 1,624, from specimens sequenced between 2006 and 2012, Table S7). Complete VP1 sequences for each genotype were evaluated, and the most conserved regions were selected as target regions for primers and probes. Multiple sets (at least three sets per genotype, AFRO and SOAS) of forward primers, reverse primers, and probes were designed to detect either of the genotypes, maintaining the same dye for the two specific probes (all probes FAM-labeled). The best-performing WPV3 primer and probe set contained two ambiguous bases in each primer and two LNAs in the probe targeting the SOAS genotype (NG54-SOAS probe). The second primer and probe set targeting the WEAF-B3 AFR genotype contained two ambiguous bases in the probe (NG50-AFR probe) and none in the primers (Table 2). The six components were premixed and used as the WPV3 assay with two probes. The assay sensitivity of 10 CCID_50_/µL at 95% LOD was identical to the previous separate WPV3-I and WPV3-II assays (Table 1; Tables S3 and S4). In the evaluation, all viruses from the CDC collection were detected, including 37 WPV3 strains from both genotypes, except for an extreme genetic outlier (collected in 1990, extinct virus, positive in the PanPV and EV assay in ITD). No cross-reactivity was observed for 125 non-WPV3 isolates, including PV type 2, type 1 viruses (Sabin-like strains, VDPV, or wild type), negative and non-polio enterovirus isolates, or IPV seed strains (Mahoney, MEF-1, Saukett), or S19 strains (Table 3). The combined WPV3 assay has significantly improved the interpretation of positive amplification curves due to the reduced background and the absence of Sabin 3 cross-reactivity. No external evaluation of the assay was conducted due to the eradication status of WPV3.

**TABLE 2:** Real-time RT-PCR assay primers and probes using LNA technologies (<nucleotide>) for the detection of wild-type 3 poliovirus assay (WPV3 assay), Sabin 1 (VDPV1 assay), and Sabin 3 (VDPV3 assay), and overhang sequences are underlined

Target (assay)	Oligo name (orientation)	Sequence (5′ to 3′)	LNA^*a*^ at nucleotide	Conc.	Genome position
WPV3 (WPV3)	NG48-WPV3-SOAS (sense)	ATGGRCATGCTCTYAACCAAG	N/A	10	2910–2930
	NG54-WPV3-SOAS probe^*b*^	T <LNA A><LNA C>CAAATAATGTACAT	2nd (A), 3rd (C)	1	2933–2949
	NG40-WPV3-SOAS (antisense)	TATGGGACAGATATTCGGGCTG	N/A	10	3042–3063
	NG35-WPV3-AFR (sense)	GGGACGATTACACCTGGCAA	N/A	10	2979–2998
	NG50-WPV3-AFR probe^*b*^	CTTTGCYAAAGTGCCSTTGA	N/A	5	3103–3122
	NG37-WPV3-AFR (antisense)	TCCCCCACTTGATCATTGGC	N/A	10	3131–3150
Sabin 1 (VDPV1)	NG6-VDPV1 (sense)	AATAAATCATAATGCGTGGCCATTATAACCGT	N/A	2.5	2735–2754
	NG24-VDPV1 probe^*b*^	A <LNA T> T <LNA C><LNA T>TGGTGGA <LNA A> G	2nd (T), 4th (C), 5th (T), 13th (A)	2	2779–2766
	NG9-VDPV1 (antisense)	AATAAATCATAATTCCTCCGTAACTGGACAGT	N/A	2.5	2822–2841
Sabin 3 (VDPV3)	S3-VDPV3-flap (sense)	AATAAATCATAACATTTACATGAAACCCAAAC	N/A	5	3276–3295
	S3-VDPV3-probe^*b*^	AGGAACAACTTGGAC	N/A	5	3360–3374
	S3-VDPV3-flap (antisense)	AATAAATCATAATGGTCAAACCTTTCTCAGA	N/A	5	3382–3400

^*a*^ LNA = locked nucleic acid is a nucleotide analog; Conc. = Concentration (pmol/µL in each reaction); N/A : not appliable.

^*b*^ All probes were labeled with 6-FAM (5′ end modification) and quenched with Black hole quencher 1 (3′ end modification) proprietary to Biosearch Technologies. IUAC: R = A or G; All target sequences are in VP1 and for the WPV3 and VDPV3 assays positions are labeled relative to human poliovirus 3 (Sabin 3, Acc. no. AY184221), and for the VDPV1 assay, human poliovirus 1 (Sabin 1, Acc. no.: AY184219). Overhang sequence underlined in primers NG6-VDPV1, NG9-VDPV1, S3-VDPV3-sense, and S3-VDPV3-antisense. All primers and probes were synthesized by Qiagen and purified by HPLC (probes > 98% purity) and standard desalting (primers > 95% purity).

**TABLE 3:** Overall results for each new assay versus the expected results with virus panel of isolates covering serotypes and genotypes (in-house assessments) with specificity and sensitivity^*d*^

New ITD assays (version 6.1) and VDPV 6.0		Expected results		Analytical sensitivity	Analytical specificity
Assay target (number of viruses tested)		Positive	Negative		
WPV1^*a*^ (*n* = 187)	Positive	31	2	100%	98.72%
	Negative	0	154		
WPV3 (*n* = 184)	Positive	32	0	96.97%	100%
	Negative	1	151		
nOPV2 (*n* = 206)	Positive	21^*b*^	0	100%	100%
	Negative	0	185		
VDPV1 (Sabin 1) (*n* = 243)	Positive	24	0	100%	100%
	Negative	0	219		
VDPV3 (Sabin 3) (*n* = 201)	Positive	36	1^*c*^	97.3%	100%
	Negative	0	164		

^*a*^ Assay specific to circulating genotype; WPV1 AFR genotype no longer detected (WEAF-B1).

^*b*^ nOPV2 strains to evaluate the nOPV2 assay (candidate 1b, *n* = 13, and 2A, *n* = 1).

^*c*^ VDPV3: This isolate was screened in the Sabin 3 assay and was late positive (Ct = 32.1, Sabin-like 3). The VDPV3 ver. 5.2 assay showed a false-negative result due to the lower assay sensitivity.

^*d*^ The VDPV3 version 6.0 detected it due to improved sensitivity now matching the Sabin 3 assay result.

### Developing a new VDPV1 assay using LNA bases and primer overhangs

LNA-designed probes typically used for single-nucleotide polymorphism (SNP) genotyping were applied to discriminate Sabin from mutated sequences (i.e., VDPV) (31). In the re-design of the VDPV1 assay, a new probe using four LNAs maintained the target site at amino acid position 99 of the VP1 but resulted in decreased fluorescence signal over the previous VDPV1 assay hindering interpretation of low signals (probe with LNAs in VDPV1 ver. 5.2). To boost the fluorescence signal, 12-base pair AT-rich overhangs were added to the forward and reverse primers (sequence of overhang: 5′-AATAAATCATAA) that are noncomplementary to the target region at the 5′ end (Table 2, VDPV1 assay) (14). The VDPV1 test performance was assessed empirically to optimize concentrations for flanking primers at 2.5–10 pmol/µL each while maintaining 2 pmol/µL probe (NG24-VDPV1) per reaction. Initially, an undesired background signal increased for negative isolates (at higher concentrations with primers at 10 pmol/µL, probe at 2 pmol/µL), necessitating adjusting the components that caused the increase. Additionally, the NG24-VDPV1 probe was evaluated against a subset of virus isolates previously characterized as Sabin 1-like to test sensitivity to Sabin 1 (target) and VDPV1 to test specificity (negative in assay = presence of VDPV1)

For the Sabin-like 1 isolates with low concentrations (i.e., high CT), version 6.0 greatly increased fluorescence intensities, boosting the signals after the addition of the overhang to flanking primers. The new VDPV1 version 6.0 maintained the same 95% LLOD (LOD95) at 10 CCID_50_/µL, and CTs were slightly lower with the new primer set (VDPV 6.0, LOD95 mean CT = 33.64, SD 1.3; *n* = 20) in contrast to version 5.2 (mean CT = 34.17, SD 1.05; *n* = 20, Table 1). Primer sets were tested with 243 PV isolates, including CDC-sequenced VDPV1 isolates (*n* = 11) and outbreak isolates (*n* = 24). The assay had 100% sensitivity for the target between the VDPV1 assay’s version 5.2 and 6.0 with insignificant CT value differences for Sabin 1 isolates (Table 3). RNA transcripts from previously misidentified VDPV1 sequences were designed and generated before VDPV1 isolates were available through GPLN partners, allowing early assay validation. Additional parallel testing of the new VDPV1 assay showed 100% concordance when testing 26 RNAs extracted from PV virus-spotted FTA cards isolated between 2019 and 2023 (*n* = 3 positive, *n* = 23 negative), and 9 isolates from VDPV1 outbreaks between 2018 and 2020 (*n* = 6 positive, *n* = 3 negative) (Table 4). Thirteen VDPV1 isolates are shown in the false-negative category (previously missed by version 5.2) but sequenced as VDPV1 could now be confirmed as VDPV1. The assay was within the performance characteristics (concordance 98% or greater).

**TABLE 4:** Two-by-two contingency comparisons of the VDPV1 real-time RT-PCR assay performance with virus isolates previously characterized through ITD and sequencing (in-house)^*a*^

*N* = 303^*b*^	Old VDPV1 assay (version 5.2)	
New VDPV1 assay (version 6.0)	Positive (VDPV1)	Negative (non-VDPV1)
Positive (VDPV1)	41	13
Negative (non-VDPV1)	0	249

^*a*^ Results for the new assay considered positive for correctly identifying VDPV1, or negative for failing to identify VDPV1 (*n* = 1) and including all other polioviruses (*n* = 223), non-polio enteroviruses (*n* = 15), and other CPE-positive viruses that were not further characterized (*n* = 10).

^*b*^ Results from PCR screening of 303 virus isolates are shown. Thirteen VDPV1 isolates are shown in the false-negative category (previously missed by version 5.2) but sequenced as VDPV1. One VDPV1 isolate that was not detected by either method is shown in the true-negative category. Sensitivity: 100%, specificity: 95.04% (PVP: 75.93%, PVN: 100%); accuracy: 95.71%, McNemar χ2 with continuity correction *P* = 0.002.

### Improving the VDPV3 assay performance with primer overhangs

The VDPV3 assay targets the antigenic region in the VP1 protein sequence at amino acid 290 in Sabin 3 viruses. The VDPV3 version 5.0 had been modified in 2010 and had a 100 times lower sensitivity than the Sabin 3 target (Quadruplex EV + Sabin assay, Tables S2 and S5), which could result in false negatives (falsely identifying VDPV3) for low-concentration samples that are Sabin 3 like (3, 32). Adding the 12-bp overhang to both the sense and antisense primers at the 5′ end (i.e., AATAAATCATAA) improved the sensitivity of the VDPV3 assay, while probe sequences remained the same (Table 2; Fig. S2; Table S2). The primers and probes were tested empirically to optimize their concentration for this new design (5, 5, 5 pmol/µL each per reaction) (Table 2). The results of the modified assay matched 99.57% with VDPV3 version 5.2 (without 12-bp overhang) and detected one more SL3 virus isolate that was missed before (Table 3). When tested with limiting dilutions of standardized Sabin 3 virus isolates, the modified VDPV3 assay had a mean CT of 31.30 (SD 0.53, *n* = 20) in comparison to CT 37.4 (SD 0.5, *n* = 20) at the lowest detectable concentrations (1 CCID_50_/µL). The VDPV3 assay version 6.0 increased its sensitivity by 10-to 100-fold, with the 95% LOD now at 1 CCID_50_/µL and Sabin 3 RNA transcripts (Fig. S2; Table S5). When testing the new VDPV1 and VDPV3 assays with their respective serotypes IPV seed strains (i.e., Mahoney and Saukett), they were assay negative as expected, as well as MEF-1 (type 2). Viral isolates collected in an outbreak in Israel were also tested for assay performance (i.e., VDPV3, Table 3).

### Piloting new assays in the GPLN

The VDPV1 and VDPV3 assays were sent to piloting labs for parallel testing of former assays and the new assays on the same PCR plate with the same set of samples identified as VDPV1 or VDPV3 through VP1 sequencing. Pilot testing was performed by the specialized global laboratory Institute Pasteur (IP), France (PV Type 1, *n* = 15; PV Type 3, *n* = 16), the regional reference labs including the NIH-Pakistan (PV Type 1, *n* = 19; PV Type 3, *n* = 21), and the National Institute of Communicable Diseases (NICD), South Africa with 30 recent VDPV1 and VDPV3 isolates or mixtures of Sabin like 1 and Sabin like 3. Assay performance was very high, with results being 100% concordant in the VDPV1 assay with expected results from sequencing virus isolates and 98.3% in the VDPV3 assay (Table S6). The Ct values were not significantly different when compared side by side with the old version of the assay.

### Development and design of the novel OPV2 assay

The nOPV2 duplex assay was released for use as a real-time PCR kit in April 2020 for distribution in the GPLN that performed AFP testing in regions with nOPV2 usage. The assay was initially thought to be used in a limited setting but became part of the ITD kit for screening for nOPV2 viruses, making it ITD version 6.0 in October 2023 (Fig. S4). The assay was developed based on the vaccine-coding sequences of candidate nOPV vaccine viruses (candidates 1b and 2A) with the nOPV2-1b targeting the Cis-replicating element (CRE) in the untranslated region at the 5′ end of the genome (CRE-5′ UTR, Table 5) (12, 26). The second probe (probe 2A) targeted the VP1 gene region for the 2A candidate (not used in the selected vaccine), and that assay component has been removed from ITD version 6.1. The singleplex nOPV2 LLOD 95% has slightly improved for the singleplex assay to 0.1 CCID_50_/µL (against nOPV2) with a mean CT value of 33.81 (SD 1.01, 20 replicates). The nOPV2 assay has no cross-reactivity with type 2 viruses that are not nOPV2 (Table 3).

**TABLE 5:** New primer and probes for WPV1/Qβ duplex real-time RT-PCR assay to detect emergent wild-type 1 poliovirus (WPV1-2024) with overhang sequences in WPV1 primers (underlined), nOPV2 singleplex assay’s primers and probe for the detection of nOPV2 vaccine with genome region and concentration per reaction^*a*^^,^^*e*^

Target (assay)	Oligo name (orientation)	Sequence (5′ to 3′)	5′ Fluorophore	3′ Quencher	Conc. (pmol/µL)	Genome position
WPV1^*d*^ (WPV1/Qβ)	WPV1-NG63 (sense)	AATAAATCATAATCAGCCTCCAGAGATGCTCT			10	2541–2560
	WPV1-NG73 Probe	GAAACGGGAGCCACGAACCC	FAM	BHQ-1	5	2622–2641
	WPV1-NG74 (antisense)	AATAAATCATAACGGTGTC YGAGGGAACTAG			10	2643–2661
Qβ (WPV1/Qβ)	Qβ-AN971 (sense)	GATGTYACTCCCTTTTACATACG			5	2083–2105^*f*^
	Qβ- AN974-Probe	CCACCGATATA SGTTATTCAAAACC	Quasar670^*b*^	BHQ-3^*c*^	4	2139–2163
	Qβ-AN972 (antisense)	GGGCYCTAGGATCCCATAC			5	2179–2197
nOPV2^~^ (nOPV2)	S2-1B (sense)	ACTTAGAAGCACGAAACCATTAA			5	104–126
	S2-1B Probe (sense)	TAGAGCAAACACCGTATAGAGCCA	CY5	BHQ-2	5	143–166
	S2-1B (antisense)	CCCCCTTCTATTGAACTTGTA			5	180–200

^*a*^ The Qβ bacteriophage target primer and probe are identical in sequence but are labeled with Cy5 and different concentrations (original publication [6]).

^*b*^ Quasar670 is equivalent to Cy5.

^*c*^ BHQ = Black hole quencher (BHQ-1, BHQ2, and BHQ-3) is proprietary to Biosearch Technologies.

^*d*^ Degenerate nucleotides follow the common International Union of Pure and Applied Chemistry (IUPAC) system.

^*e*^ Conc. = concentration (pmol/µL in each reaction), overhang sequence is underlined in primers WPV1-NG63 and WPV1-NG74. Reference genome WPV1 Acc. no.: PX448531. ~nOPV2 assay designed in 5′ nontranslated region-cis replicating element (NTR-CRE); Acc. no.: MZ245455 nOPV2.

^*f*^ Based on genome KC137661.1 Enterobacteria phage Qβ.

### Updating the duplex wild-type 1 PV assay

The WPV1 primer and probes were designed to exclusively detect the South Asian (SOAS) genotype isolates from 2018 to 2024 using the VP1 gene sequence analysis (no longer detects the extinct WPV1 genotype West Africa [WEAF-B1] that was previously covered) (4). The VP1 sequences of 1819 viruses were aligned, and the consensus sequence with the most conserved region was chosen for the assay design (as described before in the WPV3 assay design section, Table S7). Multiple sets of primers and probes were initially screened with RNA transcripts of the circulating genotype (VP1 sequence like WPV1-SOAS). After several failed attempts with cross-reactivity when tested with WPV3 or Sabin 1 isolates and low signals with the desired target (recent WPV1 isolate), additional primer and probe sets were designed. The best-performing WPV1 assay combination was selected, and primers and probes were empirically evaluated with concentrations of 1, 2, and 5 pmol/µL (WPV1-NG73 probe) and 5, 8, and 10 pmol/µL (WPV1-NG63 sense and WPV1-NG74 antisense). The signal was improved using the 12-mer AT-rich overhangs on both forward and reverse primers to maintain specificity and sensitivity (Table 5). After the WPV1 assay performance was satisfactory, it was combined with the Qβ bacteriophage component, maintaining concentrations at 5 pmol/µL for primers (sense and antisense) and 4 pmol/µL for the Qβ fluorescence-labeled probe. The final concentrations for both assays were determined by balancing the best results with low or no background in both assay targets. The WPV1/Qβ duplex assay was tested with the same Qβ probes labeled either with Cy5 or with Cy3 (Cy3 dye used in duplex WPV1/Qβ ITD version 6.0, described before [6]) using WPV1/Qβ RNA transcripts as templates. The dye Cy5 was selected for the probe due to its improved overall fluorescence in the Qβ component, making interpretation easier while maintaining the sensitivity of the Duplex WPV1/Qβ assay (Fig. S1 and S4).

### Piloting WPV1 assay in an endemic region

The new duplex WPV1/Qβ assay version 6.1 was sent to the GPLN partners at NPL-Pakistan for pilot testing with the largest and most recent WPV1 virus collection. In total, 236 WPV1 isolates from environmental surveillance samples (i.e., wastewater) and AFP stools collected in 2025 were tested side by side with version 6.0 (WPV1/Qβ). Pilot testing by the NPL-Pakistan lab showed high concordance between both assays when testing with the endemic WPV1 SOAS genotype (*n* = 233 positive/*n* = 236 total tested and expected, sensitivity 98.73%) (Table 6). The genotype-specific sensitivity version 6.1 assay improved slightly compared to the old assay, with three more detections of endemic WPV1 strains found in the region in 2023 and onward. The mean CT value decreased by ~10 from an average CT of 28.04 (SD 1.86, *n* = 233) for version 6.0 to CT 18.51 (SD 1.27, *P* < 0.0001; *n* = 236) for the WPV1/Qβ version 6.1, but the overall analytical sensitivity stayed at LOD 10 CCID_50_/µL (Table 1). The specificity and sensitivity from piloting (*n* = 236 isolates) and in-house testing (*n* = 187) were 94.3% and 96.6%, respectively. The Qβ target was negative for all tests, indicating 100% specificity to Qβ without cross-reactivity to poliovirus or non-polio-enterovirus isolates (*n* = 187). One isolate was confirmed to be a Sabin-like 3/WPV1 mixture with extinct AFR WPV1 genotype. A WPV2/WPV1 mixture that was negative in the new WPV1/Qβ duplex assay was isolated from a specimen collected in 1999 and had no sequencing information available in the database. The IPV strains were negative in the new WPV1/Qβ duplex assay.

**TABLE 6:** Pilot study performed by Pakistan’s National Polio Laboratory in a 2 × 2 contingency comparison of WPV1 assay duplex with Qβ (version 6.0) and newly designed WPV1/Qβ version 6.1^*a*^

*N* = 236^*b*^	Number of isolates with expected result from WPV1/Qβ duplex version 6.0	
WPV1/Qβ version 6.1^*c*^	Positive	Negative
Positive	233	3
Negative	0	0

^*a*^ Isolates from acute flaccid paralysis cases or environmental surveillance testing collected between 2022 and 2025.

^*b*^ Results from a total of 236 isolates are shown. Any WPV1 isolate that was tested was confirmed through Sanger sequencing of the VP1 gene (33).

^*c*^ Sensitivity: 98.73%, specificity:0% (PVP: 100%, PVN: 0%); accuracy: 98.73%.

### Assessment of assays on network cyclers and with S19 and novel OPV strains

The five assays were assessed with Quant Studio 5 and 6 Flex, Bio-Rad CFX Opus, and Rotor Gene Q cyclers, showing 99.5%–100% concordance in their performance when comparing the assay-by-assay results with 184 viruses, three IPV seed strains (Mahoney, MEF-1, and Saukett), and their respective reference strains. All ITD/VDPV assays were tested under run conditions using an ABI 7500 and performed as expected. The evaluation included S19 strains of each of the vaccine seed strains and IPV seed strains, which showed great sensitivity and specificity as expected. The vaccine strains used in clinical trials (nOPV1 and nOPV3) were tested and are positive for the following assay targets: EV, PanPV, Sabin 1 (nOPV1 strain only) or Sabin 3 (nOPV3 strain only), and nOPV assay (both nOPVs share their genetic backbone with nOPV2 viruses). Both VDPV1 (nOPV1) and VDPV3 (nOPV3) assays are positive when tested with their respective serotype strains, as they share the same primer-binding regions as Sabin viruses.

## DISCUSSION

Molecular assay development for polioviruses requires highly sensitive methods that can balance sensitivity while maintaining the specificity needed for the serotypes that are detected through real-time RT-PCR. The most recent advances in molecular assay design reported on the improved PanPV assay with a double-quenched probe in 2021, followed by the duplexed assay detecting WPV1 and the Qβ bacteriophage in 2024 (5, 6). Assay design in an eradication environment is particularly challenging due to the limited availability of suitable polioviruses to include in the development and validation. Several factors complicated the development, such as the need to maintain the same run method for any new assay that would be included in the ITD/VDPV suite, compatibility with any of the seven validated thermocyclers in the network, and the need for evaluating assays with virus isolates that require containment. The design goals were to reduce the number of assays (WPV3), increase specificity for outdated assays (VDPV1 and WPV1), increase sensitivity for VDPV3, and be able to detect the vaccine strain (nOPV2). The ITD suite and VDPV reflex assays were improved in preparation for a likely switch from cell culture-based screening of stool samples to direct RNA detection methods. Preliminary findings from pilot studies of possible testing algorithms were described before (34).

A previous version of the ITD included two separate assays (WPV3-I and WPV3-II) to detect different WPV3 genotypes found on the African continent (WEAF-B3) (last detected in 2009) and Southeast Asia (WPV3-B1, which stopped circulating in 2012), with WPV3 declared eradicated in 2019 (8). The two-probe design combining new FAM-labeled probes in a single WPV3 assay had very high target sensitivity and reduced cross-reactivity, as seen previously in the WPV3-II assay. LNA technology improves binding strength, specificity, stability, and mismatch detection, making it highly effective for detecting short or highly similar nucleic acid sequences (15, 16). The benefit of using an LNA-probe was to allow multiple primer pairs to function efficiently while maintaining the ITD run method i.e., annealing temperature of 50°C for the RT step. The updated WPV3 assay has been used in kits distributed to the GPLN laboratories, first released as the Poliovirus WPV3 real-time RT-PCR replacement assay and was then incorporated into ITD version 5.2 in 2020 (Fig. S4). The WPV3 assay has been successfully applied globally to any CPE-positive suspected poliovirus isolates from stools or environmental wastewater (about 10,000 isolates tested annually). It has proven to be of high value by reducing the number of assays, decreasing false-positive signals formerly seen in the WPV3-II assay, and removing challenges with interpretation due to low PCR signals. The GPEI program cannot miss any polioviruses, even though types 2 and 3 have been eradicated: the algorithm must maintain its ability to detect it in light of a containment breach with WPV3 at a vaccine manufacturer in the Netherlands (35).

The ITD assay suite follows a specific testing algorithm that was modified in 2021 to account for the nOPV2 assay and detailed results for downstream VP1 sequencing (Fig. S3 and S4). In ITD version 6.0, the number of assays increased to 6, adding the duplexed nOPV2 assay. In 2025, the second component of the nOPV2 assay was removed, keeping the vaccine virus (formerly candidate 1b) target as a singleplex assay due to the wide use of nOPV2 vaccines. The nOPV2 assay has no cross-reactivity with Sabin 2 viruses, but the nOPV2 strain has recombined with other polioviruses (Sabin) or species *EV Coxsackiepol,* restoring the CRE in the 2C genomic region (36, 37). Recombination events can lead to a loss of the genomic region targeted by the nOPV2 assay (5′-UTR-CRE) and lead to a false-negative result for the nOPV2 target. This makes the distinction between Sabin 2-derived VDPV2 (VDPV2) and nOPV2-derived VDPV2 (VDPV2n) impossible through real-time PCR using ITD alone, requiring full-genome sequencing for virus identification (38). Any recombinant nOPV2-derived strain would still be detected by the Sabin 2 target in the EV/Sabin assay and the PV2 assay designed to bind a different region of the VP1. Any type 2 result would automatically trigger VP1 sequencing; however, the extended window to cover the 5-UTR would need to be included in the target amplification for any type 2 since OPV2 has been dropped.

The VDPV targets in the genome region of the VP1 protein are known to encode amino acids in surface determinants that undergo strong selection during replication in the intestine (31, 39). Mutation of nucleotides in that region led to failure of the VDPV1 assay, as in the 2018 cVDPV1 outbreak where isolates were misidentified as Sabin 1 (11). The VDPV1 test was designed to maximize specificity with LNA-probe technology and targeted outbreak VDPV1 sequences. In the development phase, the number of LNAs in the probe was increased from 2 to 4 to improve performance because more mutations occurred at amino acid position codon 99 (underlined in sequence 5′-A**T**T**CT**TGGTGGA**A**G-3′) encoding the antigenic site of Sabin 1. The VDPV1 assay had increased specificity to detect the VDPV1 isolates, although the probe length was reduced from 20 to 14 nucleotides. Due to its high binding efficiency, the probe concentration was optimal at 2 pmol per µL. The new assay can discriminate Sabin 1 strains from VDPV1 from Papua New Guinea (RNA transcripts) as well as isolates from the in-house collection (*N* = 208) that the previous version of the assay missed (VDPV kit version 5.2 released in GPLN in 2020). In addition, 12-mer AT-rich overhangs were applied to primers in VDPV1 (5′-AATAAATCATAA-primer sequence-3′) and increased the fluorescent signal. Pilot testing with polioviruses isolated from environmental samples (e.g., Sabin 1-like) and the 184 virus isolates showed 100% concordance, and the modified assay has been vetted and used by the GPLN partners since its release in 2024 (VDPV1 in version 6.0, Fig. S4).

The second reflex assay in the VDPV kit is the VDPV3 assay targeting Sabin 3 in amino acid region 285–290 of VP1. The previous version of the VDPV3 assay was less sensitive for the Sabin 3 target (in the quadruplex EV/Sabin assay) because of different primer binding regions, and some Sabin 3 isolates with low viral content were missed in the VDPV3 assay. To increase the total fluorescent signal through larger amplicon size, better probe hybridization, and to enhance amplification yield, overhangs of 12-mer sequences were added to both the sense and antisense primers (5′-AATAAATCATAA-primer sequence-3′) to stabilize the reaction (14). Overhangs were shown to improve sensitivity in the VDPV3 assay by 100-fold and with an LOD of 10 CCID_50_/µL matching the Sabin 3 target in the quadruplex assay.

The WPV1 primer and probes were updated due to genetic drift leading to false-negative samples in 2022 (albeit WPV1 was still detected in generic EV and PanPV assays, and through the cross-reactive PV2 assay). Because of its specificity, 14 WPV1 isolates that belong to genetically diverse and extinct WPV1-AFR genotype previously detected by ITD version 6.0 were negative in the new assay but still found through less specific assays like the PanPV and EV assays (Table 3; Table S2). The sensitivity stayed at 10 CCID_50_/µL, but CT values were lower by adding 12-mer overhangs to the WPV1 primers (sense and antisense) for the WPV1/Qβ duplex (Fig. S1, Pilot study with Pakistan). The Qβ bacteriophage target interpretation significantly improved with the Cy5-labeled probe performing well with the bacteriophage and extracted RNA while the established cutoffs used in direct detection could be maintained (6).

A limitation of the ITD suite and VDPV real-time RT-PCR method is that they are not appropriate as stand-alone tests (i.e., single-assay use versus using the complete suite) and require the redundancy of the testing algorithm with VP1 sequencing to define genotypes (Fig. S3). The sensitivity of the assays varies; thus, it is necessary to run ITD in parallel, followed by the two reflex assays (if Sabin 1 or 3 is detected) to not miss polioviruses of programmatic importance (VDPV and WPV). If a specific genotype assay fails due to mutations in the probe-binding site, sequencing analysis resolves these drifted variants (i.e., wild-type 1 or VDPVs); or immunodeficient VDPV (iVDPV), that are highly divergent immunodeficient viruses from patients who have prolonged infections after exposure to OPV (40, 41). The ITD 6.1 algorithm works in conjunction with extended genetic sequencing, including the 5′UTR-region, which could be critical for identification of nOVP2 double recombinants as seen in VDPV2n. It was difficult to include more recent viruses in the evaluation because WPV3 has been eradicated, the endemic region has very few AFP cases (most WPV1 detections in environmental surveillance), and VDPV isolates are very rare (type 1) or have strict containment with labs destroying isolates (e.g., VDPV3). The CDC in-house viruses were collected between 1999 and 2010, many of which do not circulate anymore (such as WPV3 and WPV2 genotypes), and were updated by adding recent WPV1, VDPV1, VDPV3 isolates, and novel vaccine strains. Maintaining the ability to test for extinct viruses was exemplified when vaccine production facilities had breaches infecting workers or when polioviruses were imported into polio-free regions in the 2022 outbreak of VDPV2 in the United States (42, 43). Recently, wastewater testing found wild-type 1 in Germany, and VDPV3 in French Guiana in South America, raising concerns for under-immunized populations in polio-free regions and showing the importance of continued AFP surveillance globally (44, 45).

Pilot testing in several GPLN labs with access to currently circulating viruses with WPV1/Qβ duplex, VDPV1, and VDPV3 assays assisted in the evaluation and showed the successful application of the assays outside of CDC. The evaluation of seed strains used in vaccine production was helpful but does not replace the large genetic variety observed during outbreaks. Even though AFP cases caused by wild-type 1 have reached a new low, vaccine-derived PVs, mainly caused by type 2, are on the rise in OPV-using regions (7). Future strategies for poliovirus testing should reduce the number of PCRs, and adaptation of existing schemes, i.e., screening with generic assays first, followed by more specific assays as reflex tests. Study findings to detect polioviruses from total nucleic acid are promising with advanced extraction methods that could eliminate cell culture isolation for most regions free of circulating poliovirus (34). In a poliovirus surveillance landscape where most specimens test negative, pre-screening with the highly sensitive and specific real-time PCR assays presented here provides the Global Polio Eradication Program with a critical and powerful tool.

## SUPPLEMENTAL MATERIAL

Supplemental figures and tables jcm.00658-26-s0001.docx Fig. S1 to S4 and Tables S1 to S7.

ASM does not own the copyrights to Supplemental Material that may be linked to, or accessed through, an article. The authors have granted ASM a non-exclusive, world-wide license to publish the Supplemental Material files. Please contact the corresponding author directly for reuse.
