# Supplementary material for: Advanced molecular poliovirus diagnostics across multiple real-time PCR platforms with locked nucleic acids and primer overhangs
Source: J Clin Microbiol. 2026 Sep 16;64(10):e00658-26. doi: 10.1128/jcm.00658-26 (PMC13712947; doi:10.1128/jcm.00658-26)
Supplement: Supplementary file 1 [file jcm.00658-26-s0001.docx]

**Supplement “Advanced Molecular Poliovirus Diagnostics Across Multiple Real-Time PCR Platforms with Locked Nucleic Acids and Primer Overhangs”**

**Table S1**. Summary of poliovirus, non-polio enterovirus and non-enterovirus isolates by serotype and genotype tested at the polio laboratory at CDC, Atlanta (modified from reference [1])

| **Serotype ^a^** | **Classification ^b^** | **Specimen No. (*n* = 184)** |
| --- | --- | --- |
| PV1 | Sabin | 10 |
|  | VDPV | 11 |
|  | WEAF-B1 | 15 |
|  | SOAS | 22 |
| PV2 | Sabin | 15 |
|  | VDPV | 16 |
|  | Wild | 10 |
| PV3 | Sabin | 12 |
|  | VDPV | 14 |
|  | WPV AFR | 13 |
|  | WPV SOAS | 20 |
| Non-polio enterovirus and Negative (non-enterovirus) | NPEV^c^ | 15 |
|  | Negative^d^ | 11 |

^a^PV: poliovirus with number identifying serotype 1, 2, or 3.

^b^VDPV, (vaccine-derived poliovirus); West Africa-B1 genotype (WEAF-B1); South Asia genotype (SOAS); Wild poliovirus 3 isolates from African region, Wild poliovirus from South Asia region (WPV SOAS). Wild poliovirus 2 isolates were received from Asia, the Middle East, Africa, and the USA between 1999 and 2009;

^c^NPEV, non-polio enterovirus (coxsackievirus A4 [CV-A4], CV-A9, CV-A21, coxsackievirus B4 [CV-B4], CV-B5, echovirus 6 [E-6], E-9, E-11, E-12, EV-A71, EV-D68, Human Rhinovirus 47)

^d^Virus isolates with CPE but negative in ITD screening assays (non-enterovirus)

**Table S2.** Limit of detection for Poliovirus rRT-PCR ITD 6.0 assays (^PanPV assay 1). The additional LOD data using the new purified WPV1 SOAS-2024 virus (bolded) was tested in the Quadruplex EV/Sabin assay described in 2018 [2]. Salk Inactivated poliovirus (IPV) strains serotype 1 (Mahoney) and serotype 3 (Saukett) were positive in both EV and PanPV assays. Mahoney was positive in the Sabin 1 assay and Saukett was positive in the Sabin 3 assay.

| **Assay name** | **Gene Target** | **Polio Reference virus strain** | **LOD 95%**  **(CCID_50_ 1 µl per reaction)** | **Average Ct (lowest dilution 19/20 replicates detected)** |
| --- | --- | --- | --- | --- |
| Quadruplex EV/Sabin | EV | Sabin 1 | 10 | 34.5 |
|  |  | Sabin 2 | 10 | 34.1 |
|  |  | Sabin 3 | 1 | 34.8 |
|  |  | AFR WPV1 | 10 | 36.8 |
|  |  | **SOAS WPV1*** | **10** | **32.5** |
|  |  | nOPV2-1b | 10 | 31.6 |
|  | Sabin 1 | Sabin 1 | 1 | 35.5 |
|  | Sabin 2 | Sabin 2 | 1 | 37.0 |
|  |  | nOPV2-1b | 10 | 29.7 |
|  | Sabin 3 | Sabin 3 | 1 | 32.1 |
| PanPV^ | PV | Sabin 1 | 10 | 35.7 |
|  |  | AFR WPV1 | 100 | 36.0 |
|  |  | **SOAS WPV1*** | **1000** | **31.8** |
|  |  | nOPV2-1b | 1 | 36.6 |
| PV2 | PV2 | Sabin 2 | 10 | 37.8 |
|  |  | iVDPV2 | 100 | 35.9 |
|  |  | MEF-1 | 10 | 35.3 |
|  |  | nOPV2-1b | 100 | 35.6 |

**Table S3.** Limit of Detection for WPV3 assays described here WPV3 (version 6.1) and in version 5.1 by target with two reference virus strains from plaque purified viruses described before [2].

| Template (virus plaque purified) | AFR WPV3-WEAF-B3  (Acc. no: KY941933) | | | SOAS WPV3  (Acc. no: KY941935) | | |
| --- | --- | --- | --- | --- | --- | --- |
| **Assay name** | Dilution | CCID_50_/Virus titer/ µl | Ct value | Dilution | CCID_50_/Virus titer/ µl | Ct value |
| ITD 5.1 (WPV3 I and WPV3 -II) | 10^-6 | 1.00E+00 | 36.75 | 10^-5 | 1.00E+01 | 37.48 |
| ITD 6.1 New WPV3 (dual probes) | 10^-5 | 1.00E+01 | 32.3 | 10^-5 | 1.00E+01 | 32.89 |
|  |  |  | SD (0.89) |  |  | SD (1.09) |

**Table S4.** Sensitivity of WPV3 assay compared to previous assays (WPV3-I and WPV3-II) using WPV3 virus isolates from CDC.

|  | CT values  (isolates: WPV3-AFR, n=13; WPV3-SOAS, n=20) | | | Outlier historical virus collected (Turkmenistan/1990)  (n=1) |
| --- | --- | --- | --- | --- |
| **Assay name** | Ct value  (MIN) | Ct value  (MAX) | Mean Ct | Ct value |
| ITD 5.1 (WPV3-II-SOAS assay) | 13.40 | 27.11 | 16.5 (WPV3-SOAS)  19.3 (WPV3-AFR) | 18.79 |
| New WPV3 (two FAM-probes) | 13.13 | 25.23 | 15.86 (SOAS, n=19)  14.45 (AFR, n=13) | Not detected |

**Pink – ITD 6.1 (WPV1 + Qβ probe - CY3)**

**Blue – ITD 6.1 (WPV1 + Qβ probe - CY5)**

**Black – ITD 6.0 (WPV1/ Qβ – CY3)**

**Figure S1: Cycling profile of WPV1/Qβ Duplexed assay in AB7500** with limiting dilutions of the WPV1-2024 virus in comparison to the ITD version 6.0 WPV1/Qβ assay (black/WPV1-target FAM, -Cy3) and version ITD 6.1 WPV1/ Qβ assay with either Qβ probe label Cy3 (WPV1 FAM/pink) or Cy5 (WPV1 FAM/blue). Starting concentration undiluted virus through 10^-6^ CCID_50_ per reaction with 1 µL template.

**Red – VDPV3 vers. 6.0 (with overhang)**

**Blue – VDPV3 vers. 5.2**

**Figure S2:** **Cycling profile of VDPV3 assays in AB7500** Positive control Sabin 3 RNA transcript at 1 pg through 100 ag in 10-fold serial dilution (in triplicates with 1 µl per reaction) between the VDPV3 assay 5.2 version (blue) and new VDPV3 assay (6.0 version, red).

**Table S5.** **VDPV3 assay performance** showed average Ct values when tested with Sabin 3 RNA transcript dilutions showing improved sensitivity for VDPV3 version 6.0 assay with AT-rich overhang for 1 pg, 100 fg, 10 fg, 1 fg, and 100 ag steps per reaction with 1 µl template RNA.

|  | **Average CT** per dilution step (SD of triplicates) | | | | |
| --- | --- | --- | --- | --- | --- |
|  | Undiluted | 1:10 | 1:100 | 1:1000 | 1:10,000 |
| **Assay** | (concentration: 1pg) | 100 fg | 10 fg | 1 fg | 100 ag |
| VDPV3 version 5.2 | 20.0 (0.04) | 23.7 (0.11) | 27.8 (0.22) | 31.7 (0.15) | 36.1 (0.52) |
| VDPV3 version 6.0 | 18.3 (0.06) | 21.1 (0.17) | 24.2 (0.24) | 27.5 (0.39) | 30.8 (0.5) |
| Difference CT (>3 Ct = one log) | 1.7 | 2.6 | 3.6 (p<0.0001) | 4.2 (p<0.0001) | 5.3 (p=0.0002) |

**Table S6: Pilot study** **VDPV1 and VDPV3 6.0 assay** performed by Pasteur Institute (PI), France, NICD, South Africa, Pakistan’s National Polio Laboratory in a 2x2 contingency comparison of VDPV1 assay (version 5.2, released in 2020) and newly designed assays VDPV version 6.0 (released in 2024). Any VDPV1 and VDPV3 isolate from acute flaccid paralysis cases or environmental surveillance was confirmed through Sanger sequencing in the VP1 gene with previously described methods (date of collection 2022-2025).

| N = 64 |  | **VDPV1 assay (vers. 5.2)** | |
| --- | --- | --- | --- |
|  |  | Positive | Negative |
| **VDPV1 assay (vers. 6.0)** | Positive | 37 | 3 |
|  | Negative | 0 | 27 |
|  |  |  |  |
| **VDPV3 results** | | | |
| N = 67 |  | **VDPV3 assay (vers. 5.2)** | |
|  |  | Positive | Negative |
| **VDPV3 assay (vers. 6.0)** | Positive | 59 | 1* |
|  | Negative | 0 | 7 |

**Note:***VP1 sequencing confirmed a Sabin 3-like sequence with 1 nt difference to Sabin 3 in this isolate, the assay’s better sensitivity detected this Sabin 3-like while the old assay did not. 98.5% concordance.

**Table S7.** Virus genomes used for assay design for WPV3, and WPV1 assays

| **Virus genotype** | **Assay target** | **Year of collection of sequences** | **Number of sequences in alignment** |
| --- | --- | --- | --- |
| WPV3  WEAF-B3 | WPV3 | 2006-2012 (African cluster), 2008-2009 (Angola, Nepal cluster) | 1379 |
| WPV3 (SOAS) | WPV3 | 2006-2012 (Afghanistan, Pakistan) | 245 |
| WPV1 | WPV1-SOAS | 2017-2024 (Afghanistan, Pakistan) | 1819 |
| WPV1 | WPV1-SOAS | 2015 Pakistan KY941935 | 1 |

**Figure S3.** ITD 6.1 Algorithm

**Sequence 5’UTR-complete capsid or WGS (nOPV2)**

**PanEV/Sabin-Quadruplex**

**PanPV**

**WPV1/Qβ (new)**

**WPV3**

**PV2**

**nOPV2 (new)**

**Report non-polio enterovirus**

**Sequence Complete VP1**

**Report non-enterovirus**

**All negative**

**Presumptive**

**Sabin-like**

**PanEV positive**

**Sequence Complete VP1**

Non-Sabin-like

**Report Sabin-like**

**Non-Sabin-like**

PanPV positive and Sabin negative

or any wild PV positive, any PV2,

Indeterminate, Invalid

**L20B positive cell culture**

**Other assays negative**

Sabin-like

**VDPV1**

**VDPV3**

**Figure S4. Evolution of ITD and VDPV kits (kit versions between 2018-2026)**

**References**

1.	Sun, H., et al., *Validation of a redesigned pan-poliovirus assay and real-time PCR platforms for the global poliovirus laboratory network.* PLoS One, 2021. **16**(8): p. e0255795.

2.	Gerloff, N., et al., *Diagnostic Assay Development for Poliovirus Eradication.* J Clin Microbiol, 2018. **56**(2).
